# Independent and additive effects of binge drinking and obesity on liver enzymes: a cross-sectional analysis using the Korean National Health Insurance Service data

**DOI:** 10.1093/gastro/goad074

**Published:** 2024-01-09

**Authors:** Anthony Kityo, Sang-Ah Lee

**Affiliations:** Department of Preventive Medicine, School of Medicine, Kangwon National University, Gangwon, Republic of Korea; Department of Preventive Medicine, School of Medicine, Kangwon National University, Gangwon, Republic of Korea; Interdisciplinary Graduate Program in Medical Bigdata Convergence, Kangwon National University, Gangwon, Republic of Korea

**Keywords:** obesity, binge drinking, liver enzymes, logistic regression, additive effects

## Abstract

**Background:**

Binge drinking (BD) has been associated with elevated liver enzymes, but the joint association of BD and adiposity with liver enzymes is understudied. We aimed to examine the combined association of BD and obesity with elevated liver enzymes.

**Methods:**

Data were obtained from 285,600 patients in the Korean National Health check-up program during 2009–2015. Level I BD (BD I) was defined as alcohol consumption of >60 g (men) or >40 g (women) on one occasion in the previous year. High-intensity BD (HIBD) corresponded to at least two times the BD I levels. General and abdominal obesity were defined by body mass index and waist circumference. Logistic regression was used to examine the independent and joint associations of BD and obesity with elevated alanine aminotransferase (ALT), aspartate aminotransferase (AST), and gamma-glutamyl transferase (GGT) levels. Relative excess risk (RERI), attributable proportion (AP), and synergy index (SI) were calculated to estimate the additive interaction effects.

**Results:**

The mean age was 42.1 ± 0.03 years and 50.2% were women. Elevated ALT [odds ratio (OR) 1.09, 95% confidence interval (CI) 1.02–1.16], AST (OR 1.16, 95% CI 1.11–1.23), and GGT (OR 1.84, 95% CI 1.05–1.94) were associated with HIBD. Higher odds of elevated ALT (OR 3.57, 95% CI 3.43–3.71), AST (OR 3.47, 95% CI 3.37–3.58), and GGT (OR 2.10, 95% CI 1.98–2.12) were observed in individuals with general obesity. A similar trend was observed for abdominal obesity. The RERI, AP, and SI for the interaction effect of BD and general obesity were 23%, 7%, and 13% for elevated AST levels, and 67%, 24%, and 58% for elevated GGT levels, respectively. Similar effects were observed for the interaction between BD and abdominal obesity.

**Conclusions:**

Obesity aggravated the odds of elevated liver AST and GGT levels in HIBD.

## Introduction

According to the World Health Organization (WHO), Korea ranks third in terms of alcohol consumption. In 2019, individuals aged ≥15 years in Korea consumed an average of 8.45 liters of alcohol, which is higher than the global average of 5.8 liters per person [1]. In addition to the amount of alcohol consumed, alcohol intake patterns such as binge drinking (BD) have been investigated in Korea [2]. In previous national surveys, the prevalence of BD (the consumption of at least five glasses or 60 g of *soju* in men and four glasses or 40 g in women at least once a week) was 46.3% and 9.2% in men and women, respectively [2].

BD increases the risk of mortality [3, 4], cardiovascular disease [5], acute traumatic events [6], and advanced liver disease [7]. In addition to BD, adiposity is also an independent risk factor for liver diseases [8, 9]. The prevalence of liver disease has been reported to increase simultaneously with obesity and excessive alcohol intake in the Korean population [10]. Therefore, it is plausible that the impact of excessive alcohol intake on hepatic health may be accelerated by the increasing prevalence of obesity in Korea.

The levels of liver function biomarkers such as enzymes may reflect the effects of alcohol intake and adiposity on the risk of advanced hepatic events [11–13]. A few studies have shown that BD is associated with liver disease [14, 15] and elevated liver enzymes [16, 17]. However, the joint effects of BD and adiposity on liver enzymes have not been widely reported [16, 17], especially in the Korean population. Most studies have focused on joint effects of adiposity and alcohol intake in general. Loomba *et al.* [18] found a joint association of alcohol intake and obesity with elevated serum alanine aminotransferase (ALT), but this study only used body mass index (BMI) to measure adiposity, targeted older adults, included only two liver enzymes, and did not consider BD patterns. Other studies have also investigated the joint effects of obesity and high alcohol intake on liver function markers [13, 19–23], but were conducted in European populations, and none of them considered BD or evaluated additive interaction effects.

This study aimed to examine the independent and combined influence of BD and obesity on liver enzymes using a nationally representative sample of Korean adults.

## Patients and methods

### Ethics approval

All participants provided written informed consent before participating in the study. This study was approved by the Institutional Review Board of Kangwon National University Hospital (IRB NO. KWNUIRB-2021–11-004–002).

### Study participants

This was a cross-sectional analysis of data from subscribers of the Korean National Health Insurance Service (KNHIS)—a compulsory insurance service provider managed by the government of the Republic of Korea [24]. The KNHIS covers almost the entire Korean population (97.1%) and contains data on demographic characteristics, diagnostic information, inpatient and outpatient medical claims, medical procedures, drug prescription claims, payment of insurance premiums, and mortality. In addition, subscribers are interviewed and examined annually or biennially to gather information on lifestyle characteristics, including alcohol consumption; physical characteristics, such as anthropometrics, X-ray, and blood pressure; and biochemical information, including serum lipids, serum creatinine, fasting blood glucose, and liver enzymes. Insurance subscribers who participated in the 2009–2015 health examination were included (*n *=* *460,149).

**Figure 1.**
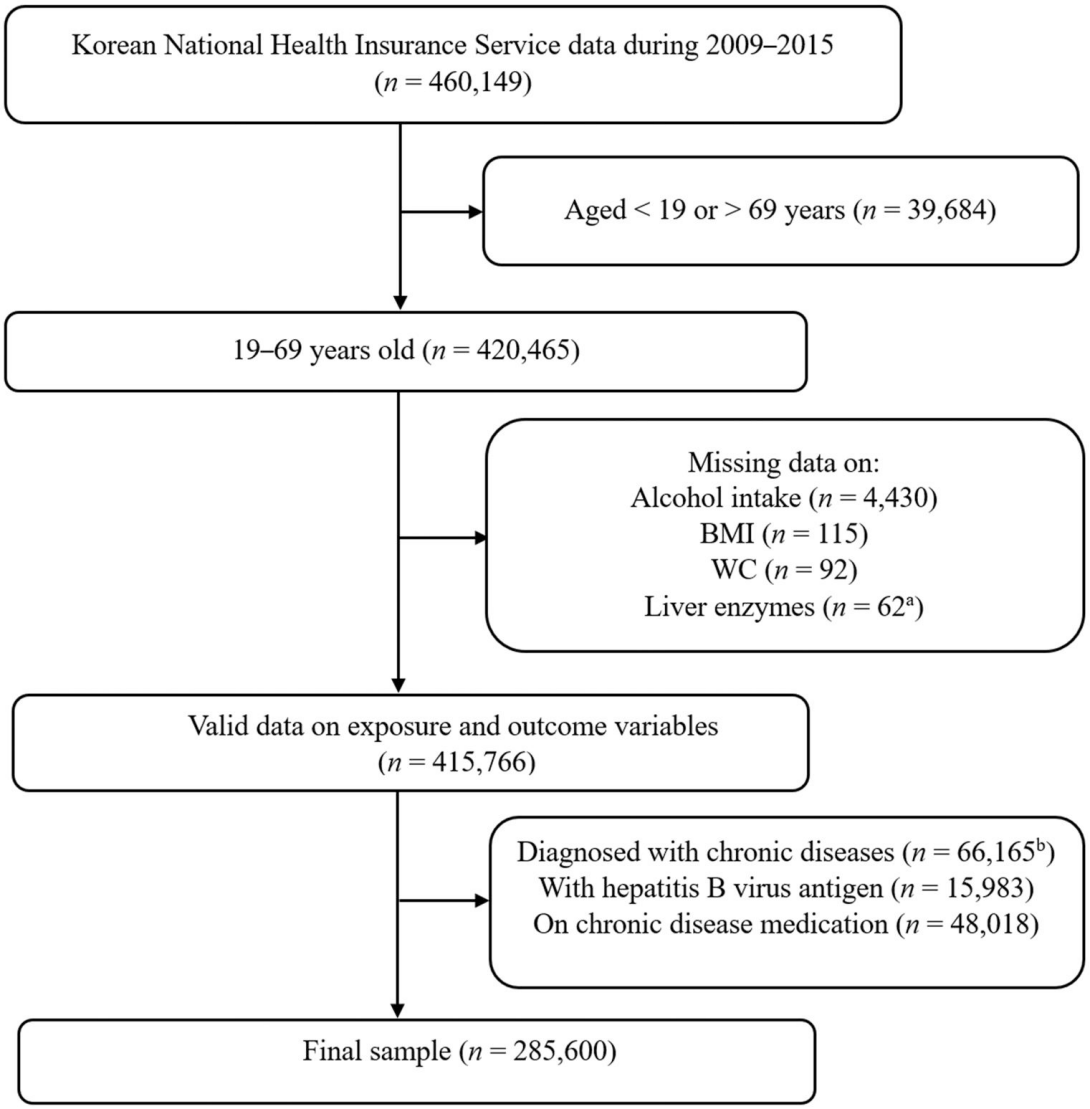
Flow chart showing selection of study participants. ^a^AST = aspartate aminotransferase, ALT = alanine aminotransferase, GGT = gamma-glutamyl transferase. ^b^Ischemic heart disease and hypertension (I10–I25), stroke (I64), heart failure (I50); diabetes mellitus (E10–E14), hepatic diseases (K70–K77); cancer (C00–C97); chronic kidney disease (N18); chronic obstructive pulmonary disease (J44); celiac disease (K90); chronic gastritis (K29, K25); Wilson’s disease (E83.0); Parkinson’s disease (G20); and Alzheimer’s disease.

We excluded individuals aged <19 or >69 years (*n *=* *39,684) and those with missing data on alcohol intake (*n *=* *4,430), BMI (*n *=* *115), waist circumference (WC, *n *=* *92), and liver enzymes (*n *=* *62). To minimize the potential effect of medication on liver enzyme levels, we further excluded participants with a medical diagnosis of cardiovascular disease, diabetes, cancer, chronic kidney disease, chronic obstructive disease, chronic gastritis, celiac disease, Wilson’s disease, and mental disorders (*n *=* *66,165) or intake of medication for these diseases (*n *=* *48,018); and those with hepatitis B virus antigen (*n *=* *15,983). Finally, we analysed 285,600 participants (Figure 1).

### Evaluation of BD

The health examination questionnaire was used to interview patients regarding (i) the number of days a person drank alcohol in a month during the past 12 months and (ii) the number of standard drinks (glasses) consumed in each drinking session. The volume of a standard drink of soju and beer, the most common alcoholic beverage in Korea, was used to compute alcohol intake. The volumes of beer and soju glasses are 220 and 50 ml, respectively. Considering the alcohol content of beer to be 4.5%, that of soju to be 21%, and 0.79 to be the specific gravity of alcohol, the alcohol content in one standard drink was computed as follows: beer, 220 × 0.045 *×* 0.79 = 7.821 g; and soju, 50 *×* 0.21 *×* 0.79 = 8.295 g. Finally, the average of the two amounts (8.1 g) was assumed to be the alcohol content of one standard drink. Alcohol intake was computed by multiplying the number of standard drinks by the alcohol content of the standard drink. BD was defined as alcohol consumption of >60 g in men or >40 g in women in the previous 12 months [25]. Binge drinkers were further classified as level I (>60 and <120 g in men or >40 and <80 g in women), level II (>120 g in men or >80 g in women), and level III BD (>180 g in men and >120 g in women) in the previous 12 months [26]. High-intensity BD (HIBD) was defined as level II or III BD [27]. Participants were classified as never drinkers, non-binge drinkers (non-BD, defined as consumption of ≤60 g of alcohol in men or ≤40 g in women in the previous 12 months), level I BD (BD I), and HIBD as defined above.

### Assessment of obesity

Height and weight were measured and accurately recorded by trained staff. BMI was calculated as weight (kg) divided by the square of height (m). BMI and WC were used as measures of general and abdominal obesity, respectively. BMI was categorized into four classes based on the WHO classification for Asian adults: <18.5, 18.5–22.9, 23.0–24.9, and ≥25.0 kg/m^2^ [28]. General obesity was defined as BMI of ≥25.0 kg/m^2^ and abdominal obesity was defined as WC of ≥90 cm in men and ≥80 cm in women [29].

### Definition of elevated liver enzymes

Elevated fasting serum levels of aspartate aminotransferase (AST), alanine aminotransferase (ALT), and gamma-glutamyl transferase (GGT) were the primary outcome variables. The following upper limits were used to define elevated ALT and GGT levels: ALT, 50 U/L in men and 35 U/L in women; GGT, 60 U/L in men and 40 U/L in women [17]. Elevated AST levels were defined as AST of >34 U/L according to the guidelines of the American College of Gastroenterologists [30].

### Statistical analysis

All data were analysed using SAS software (version 9.4; SAS Institute Inc., Cary, NC, USA) and statistical significance was defined as *P *<* *0.05. The distribution of participant characteristics according to BD intensity is presented as frequencies (percentages) for categorical variables. Continuous variables are presented as least square means accompanied by their standard errors (SEs).

Multivariable logistic regression was used to estimate the odds ratios (ORs) and 95% confidence intervals (CIs) of elevated liver enzymes according to (i) BD intensity and (ii) general and abdominal obesity. In the BD–liver enzymes analysis, models were adjusted for age, sex (male and female), smoking (never, current, and past smoker), moderate physical exercise (defined as vigorous intensity activities performed three times a week for 30 minutes or moderate walking at least five times a week for at least 30 minutes or moderate/vigorous intensity exercise at least 5 days a week lasting 30 minutes), income level (low, middle, and high classified based on insurance premiums), insurance type (Medicaid, self- and employee-insured), residence (Seoul, Gyeongi-do, metropolitan cities, and rural areas based on city codes), BMI (continuous), fasting blood glucose, high blood pressure (systolic blood pressure > 130 mmHg or diastolic blood pressure > 85 mmHg), and dyslipidemia (triglyceride levels ≥150 mg/dL or high-density lipoprotein cholesterol levels <40 mg/dL in women or <50 mg/dL in men, or total cholesterol levels ≥ 240 mg/dL or low-density lipoprotein cholesterol levels >160 mg/dL). For BMI and WC–liver enzymes analyzes, the models were further adjusted for BD intensity.

The joint effects of BD and general and abdominal obesity on elevated liver enzymes were assessed by combining variables for BMI/WC and BD into four categories: non-BD with BMI of <23.0 kg/m^2^, non-BD with BMI of ≥23.0 kg/m^2^, BD (BD I and HIBD) with BMI of <23.0 kg/m^2^, and BD with BMI of ≥23.0 kg/m^2^. The analysis of joint effects was performed after excluding participants who never drank alcohol. Three measures of additive interaction—the relative excess risk due to interaction (RERI), the attributable proportion (AP), and the synergy index (SI)—were estimated together with their 95% CIs using Hosmer and Lemeshow’s approach [31] and implemented according to [32].

## Results

### Description of participants according to BD

A total of 285,600 Korean citizens (women, 50.2%) with a mean age of 42.1 ± 0.03 years was analysed. The prevalences of elevated ALT, AST, and GGT levels were 7.9%, 15.3%, and 12.5%, respectively, and the prevalences of BD I and HIBD were 15.1% and 4.3%, respectively. About 30% had a BMI of ≥25.0 kg/m^2^ and 19.1% had abdominal obesity. Demographic, lifestyle, and clinical characteristics according to BD are shown in Table 1. Individuals in the HIBD group were more likely to be young, men, and current smokers, and engage in moderate physical exercise than those in the never drinkers. In addition, individuals in the HIBD group were more likely to be low-income earners, self-insured, live in Gyeongi (Seoul Metropolitan area), and have a high BMI. Furthermore, blood glucose levels and the proportion of elevated ALT, AST, GGT, dyslipidemia, and high blood pressure were higher in HIBD than in never drinkers (Supplementary Table S1).

**Table 1.** Demographic, lifestyle, and clinical characteristics according to BD intensity

Characteristic	Binge-drinking intensity			
	Never drinker (*n *=* *136,715)	Non-BD (*n *=* *93,536)	BD I (*n *=* *43,171)	HIBD (*n *=* *12,178)
Age, years, mean ± SE	44.7 ± 0.03	41.2 ± 0.04	37.8 ± 0.06	35.0 ± 0.11
Sex, *n* (%)				
Men	42,393 (31.0)	63,202 (67.6)	29,700 (68.2)	8,358 (68.6)
Women	94,322 (69.0)	30,334 (32.4)	13,471 (31.8)	3,820 (31.4)
Smoking, *n* (%)				
Never	108,925 (79.1)	43,449 (46.5)	15,555 (36.0)	3,368 (27.7)
current	9,821 (7.2)	15,830 (16.9)	7,174 (16.6)	2,126 (17.5)
Past	17,969 (13.1)	34,257 (36.6)	20,442 (47.4)	6,684 (54.9)
Alcohol intake, g/week, mean ± SE	0.0 ± 0.22	68.8 ± 0.27	167.3 ± 0.4	340.4 ± 0.74
Moderate physical exercise, *n* (%)	59,021 (43.2)	47,886 (51.2)	21,669 (50.2)	6,093 (50.0)
Income level, *n* (%)				
Low	34,268 (25.1)	23,522 (25.2)	11,475 (26.6)	3,544 (29.1)
Middle	52,778 (38.6)	36,567 (39.1)	17,440 (40.4)	4,822 (39.6)
High	49,669 (36.3)	33,447 (35.8)	14,256 (33.0)	3,812 (31.3)
Insurance type, *n* (%)				
Medicaid	1,580 (1.2)	691 (0.7)	375 (0.9)	137 (1.1)
Self-insured	32,571 (23.8)	23,025 (24.6)	10,662 (24.7)	2,982 (24.5)
Employee-insured	102,564 (75.0)	69,820 (74.7)	32,134 (74.4)	9,059 (74.4)
Residence, *n* (%)				
Seoul	30,354 (22.2)	22,939 (24.5)	9,751 (22.6)	2,594 (21.3)
Gyeongi-do	38,349 (28.1)	25,910 (27.7)	12,345 (28.6)	3,558 (29.2)
Metropolitan city	27,539 (20.1)	19,950 (21.3)	9,040 (20.9)	2,474 (20.3)
Rural area	40,473 (29.6)	24,737 (26.5)	12,035 (27.9)	3,552 (29.2)
Abdominal obesity, *n* (%)	29,413 (21.5)	14,762 (15.8)	7,889 (18.3)	2,636 (21.7)
BMI categories, *n* (%)				
<18.0 kg/m^2^	7,316 (5.4)	4,159 (4.5)	1,702 (3.9)	480 (3.9)
18.0–22.9 kg/m^2^	61,300 (44.8)	39,346 (42.1)	16,773 (38.9)	4,361 (35.8)
23.0–24.9 kg/m^2^	30,755 (22.5)	22,557 (24.1)	9,948 (23.0)	2,683 (22.0)
≥25.0 kg/m^2^	37,344 (27.3)	27,474 (29.4)	14,748 (34.2)	4,654 (38.2)
Fasting blood glucose, mg/dL, mean ± SE	93.6 ± 0.05	95.2 ± 0.06	95.2 ± 0.1	95.1 ± 0.18
Elevated ALT, *n* (%)	10,196 (7.5)	6,953 (7.4)	4,027 (9.3)	1,265(10.4)
Elevated AST, *n* (%)	16,470 (12.1)	15,611 (16.7)	8,793 (20.4)	2,769 (22.7)
Elevated GGT, *n* (%)	7,970 (5.8)	14,743 (15.8)	9,911 (23.0)	3,109 (25.5)
Dyslipidemia, *n* (%)	58,722 (43.0)	37,050 (39.6)	18,426 (42.7)	5,311 (43.6)
High blood pressure, *n* (%)	35,597 (26.0)	30,422 (32.5)	15,270 (35.4)	4,344 (35.7)

SE = standard error, BD = binge drinking, BMI = body mass index, BD I = level I binge drinking, HIBD = high-intensity binge drinking, AST = aspartate aminotransferase, ALT = alanine aminotransferase, GGT = gamma-glutamyl transferase.

Complete percentages are provided in Supplementary Table S1.

### Description of participants according to general and abdominal obesity

The characteristics of participants according to BMI and WC categories are shown in Table 2. Individuals with obesity or abdominal obesity tended to be older, self-insured, and rural residents compared with those with normal weight. The proportions of individuals with elevated ALT, AST, GGT, dyslipidemia, and high blood pressure were higher among individuals with general and abdominal obesity. However, individuals with the highest BMI were more likely to be men, current smokers, engage in moderate physical exercise, and be high-income earners, whereas those with abdominal obesity were more likely to be women, never smokers, and low-income earners compared with those with the lowest BMI or WC. Fasting blood glucose levels and the prevalence of elevated liver enzymes were higher among individuals with general or abdominal obesity than their counterparts (Supplementary Table S2).

**Table 2.** Demographic, lifestyle, and clinical characteristics according to BMI and abdominal obesity

Characteristic	BMI category, kg/m^2^				Abdominal obesity	
	<18.0 (*n *=* *13,657)	18.0–22.9 (*n *=* *121,780)	23.0–24.9 (*n *=* *65,943)	≥25.0 (*n *=* *84,220)	No (*n *=* *230,900)	Yes (*n *=* *54,700)
Age, years, mean ± SE	34.9 ± 0.1	40.6 ± 0.0	44.3 ± 0.1	43.7 ± 0.0	41.2 ± 0.0	46.0 ± 0.1
Sex, *n* (%)						
Men	3,235 (23.7)	48,510 (39.8)	38,189 (57.9)	53,449 (63.5)	119,888 (51.9)	23,495 (43.0)
Women	10,422 (76.3)	73,270 (60.2)	27,754 (42.1)	30,771 (36.5)	111,012 (48.1)	31,205 (57.1)
Smoking, *n* (%)						
Never smoker	10,218 (74.8)	80,902 (66.4)	37,044 (56.2)	43,133 (51.2)	136,458 (59.1)	34,839 (63.7)
Current smoker	743 (5.4)	11,129 (9.1)	9,646 (14.6)	13,433 (16.0)	28,545 (12.4)	6,406 (11.7)
Past smoker	2,696 (19.7)	29,749 (24.4)	19,253 (29.2)	27,654 (32.8)	65,897 (28.5)	13,455 (24.6)
Alcohol intake, g/week, mean ± SE	38.7 ± 0.9	52.3 ± 0.3	66.2 ± 0.4	77.7 ± 0.4	62.4 ± 0.2	62.1 ± 0.5
Moderate physical exercise, *n* (%)	5,698 (41.7)	56,535 (46.4)	32,021 (48.6)	40,415 (48.0)	110,041 (47.7)	24,628 (45.0)
Income level, *n* (%)						
Low	3,749 (27.5)	31,183 (25.6)	16,307 (24.7)	21,570 (25.6)	58,584 (25.4)	14,225 (26.0)
Middle	5,303 (38.8)	47,830 (39.3)	25,448 (38.6)	33,026 (39.2)	90,204 (39.1)	21,403 (39.1)
High	4,605 (33.7)	42,767 (35.1)	24,188 (36.7)	29,624 (35.2)	82,112 (35.6)	19,072 (34.9)
Insurance type, *n* (%)						
Medicaid	207 (1.5)	1,242 (1.0)	585 (0.9)	749 (0.9)	2,191 (1.0)	592 (1.1)
Self-insured	2,780 (20.4)	28,243 (23.2)	16,269 (24.7)	21,948 (26.1)	55,099 (23.9)	14,141 (25.9)
Employee-insured	10,670 (78.1)	92,295 (75.8)	49,089 (74.4)	61,523 (73.1)	173,610 (75.2)	39,967 (73.1)
Residence, *n* (%)						
Seoul	3,408 (25.0)	28,471 (23.4)	14,901 (22.6)	18,858 (22.4)	53,231 (23.1)	12,407 (22.7)
Gyeongi-do	3,822 (28.0)	34,545 (28.4)	18,606 (28.2)	23,189 (27.5)	65,324 (28.3)	14,838 (27.1)
Metropolitan city	2,856 (20.9)	25,098 (20.6)	13,429 (20.4)	17,620 (20.9)	47,601 (20.6)	11,402 (20.8)
Rural area	3,571 (26.2)	33,666 (27.6)	19,007 (28.8)	24,553 (29.2)	64,744 (28.0)	16,053 (29.4)
Elevated ALT, *n* (%)	303 (2.2)	4,145 (3.4)	4,499 (6.8)	13,494 (16.0)	13,102 (5.7)	9,339 (17.1)
Elevated AST, *n* (%)	459 (3.4)	8,120 (6.7)	9,824 (14.9)	25,240 (30.0)	28,419 (12.3)	15,224 (27.8)
Elevated GGT, *n* (%)	493 (3.6)	8,045 (6.6)	8,583 (13.0)	18,612 (22.1)	24,597 (10.7)	11,136 (20.4)
Fasting blood glucose, mg/L, mean ±SE	88.9 ± 0.2	91.7 ± 0.1	95.3 ± 0.1	98.5 ± 0.1	93.3 ± 0.0	98.9 ± 0.1
Dyslipidemia, *n* (%)	2,167 (15.9)	35,825 (29.4)	30,726 (46.6)	50,791 (60.3)	85,622 (37.1)	33,887 (62.0)
High blood pressure, *n* (%)	1,467 (10.7)	24,450 (20.1)	21,357 (32.4)	38,359 (45.6)	61,070 (26.5)	24,563 (44.9)

SE = standard error, BMI = body mass index, AST = aspartate aminotransferase, ALT = alanine aminotransferase, GGT = gamma-glutamyl transferase.

Completed percentages are provided in Supplementary Table S2.

### Independent association of general and abdominal obesity with elevated liver enzymes

Obesity and BD were independently and positively associated with elevated liver enzymes (Table 3). The highest odds of elevated ALT (OR 1.09, 95% CI 1.02–1.16), AST (OR 1.16, 95% CI 1.11–1.23), and GGT (OR 1.84, 95% CI 1.05–1.94) were observed among patients in the HIBD group compared with those in the non-BD group. In addition, the highest odds of elevated ALT (OR 3.57, 95% CI 3.43–3.71), AST (OR 3.47, 95% CI 3.37–3.58), and GGT (OR 2.10, 95% CI 1.98–2.12) were observed among individuals with a BMI of ≥25.0 kg/m^2^. Moreover, individuals with abdominal obesity had higher odds of elevated ALT (OR 2.70, 95% CI 2.62–2.78), AST (OR 2.64, 95% CI 2.58–2.71), and GGT (OR 1.83, 95% CI 1.78–1.89).

**Table 3.** Independent association of BD and obesity measures with elevated liver enzymes

Category	Elevated liver enzymes					
	ALT		AST		GGT	
	*n*	**OR** ^a^ **(95% CI)**	*n*	OR (95% CI)	*n*	OR (95% CI)
**Binge-drinking intensity**						
Never drinker	10,196	1.00	16,470	1.00	7,970	1.00
Non-BD	6,953	1.20 (1.160–1.25)	15,611	1.14 (1.10–1.17)	14,743	0.45 (0.43–0.46)
BDI	4,027	1.10 (1.05–1.15)	8,793	1.12 (1.08–1.16)	9,911	1.62 (1.56–1.67)
HIBD	1,265	1.09 (1.02–1.16)	2,769	1.16 (1.11–1.23)	3,109	1.84 (1.05–1.94)
**Body mass index**						
<18.0 kg/m^2^	303	0.75 (0.67–0.85)	459	0.71 (0.64–0.78)	493	0.92 (0.83–1.01)
18.0–22.9 kg/m^2^	4,145	1.00	8,120	1.00	8,045	1.00
23.0–24.9 kg/m^2^	4,499	1.66 (1.59–1.74)	9,824	1.70 (1.65–1.76)	8,538	1.34 (1.29–1.39)
≥25.0 kg/m^2^	13,494	3.57 (3.43–3.71)	25,240	3.47 (3.37–3.58)	18,612	2.10 (1.98–2.12)
**Abdominal obesity**						
No	13,102	1.00	28,419	1.00	24,597	1.00
Yes	9,339	2.7 (2.62–2.78)	15,224	2.64 (2.58–2.71)	11,136	1.83 (1.78–1.89)

BD = binge drinking, HIBD = high-intensity binge drinking, BD I = level I binge drinking, AST = aspartate aminotransferase, ALT = alanine aminotransferase, GGT = gamma-glutamyl transferase.

a Adjusted for age, sex, smoking, drinking, moderate/vigorous intensity exercise, income level, insurance, and residence, adjusted for BMI (for the binge-drinking model), fasting blood glucose, high blood pressure, and dyslipidemia.

Partially adjusted models are shown in Supplementary Table S3.

### Joint effects between obesity and BD on elevated liver enzymes

The joint and additive interaction effects of BD and general obesity on elevated liver enzymes are shown in Table 4. Compared with participants with a BMI of <23.0 kg/m^2^ in the non-BD category, those with obesity and in the HIBD group showed higher odds of elevated ALT (OR 3.88, 95% CI 3.52–4.27), AST (OR 4.04, 95% CI 3.75–4.35), and GGT (OR 3.83, 95% CI 3.56–4.13). Similarly, individuals in the HIBD group who had abdominal obesity showed the highest odds of elevated ALT (OR 3.04, 95% CI 2.74–3.36), AST (OR 3.16, 95% CI 2.88–3.45), and GGT (OR 3.62, 95% CI 3.31–3.97) than those in the non-BD category and without abdominal obesity (Table 5).

**Table 4.** Joint association and additive interaction effects of BD and BMI on elevated liver enzymes

Outcome	BD Intensity	BMI, kg/m^2^						Interaction measures		
		<23.0		23.0–24.9			≥25.0	RERI^a^ (95% CI)	AP^a^ (95% CI)	SI^a^ (95% CI)
		*n*	OR^a^ (95% CI)	*n*	OR^a^ (95% CI)	*n*	OR^a^ (95% CI)			
Elevated ALT										
	Non-BD	1,306	1.00	1,398	1.62 (1.50–1.76)	4,249	3.66 (3.42–3.91)	0.14 (–0.02–0.29)	0.05 (–0.05–0.15)	1.10 (0.99–1.17)
	BDI	664	1.13 (1.03–1.25)	754	1.84 (1.68–2.02)	2,609	3.89 (3.61–4.18)			
	HIBD	201	1.29 (1.11–1.50)	210	1.82 (1.56–2.13)	854	3.88 (3.52–4.27)			
Elevated AST										
	Non-BD	3,115	1.00	3,651	1.69 (1.60–1.78)	8,845	3.48 (3.32–3.65)	0.23 (0.11–0.34)	0.07 (0.01–0.14)	1.13 (1.10–1.21)
	BDI	1,499	1.12 (1.05–1.20)	1,897	1.91 (1.79–2.03)	5,397	3.84 (3.64–4.05)			
	HIBD	446	1.32 (1.19–1.47)	522	1.88 (1.69–2.10)	1,801	4.04 (3.75–4.35)			
Elevated GGT										
	Non-BD	3,855	1.00	3,683	1.25 (1.19–1.32)	7,205	1.89 (1.81–1.99)	0.67 (0.55–0.76)	0.24 (0.18–1.28)	1.58 (1.44–1.72)
	BD I	2,152	1.52 (1.43–1.61)	2,340	2.1 (1.98–2.24)	5,419	3.19 (3.03–3.36)			
	HIBD	577	1.74 (1.58–1.93)	652	2.29 (2.06–2.54)	1,880	3.83 (3.56–4.13)			

BD = binge drinking, HIBD = high-intensity binge drinking, BMI = body mass index, RERI = relative excess risk due to interaction, AP = attributable proportion, SI = synergy index, BD I = level I binge drinking, AST = aspartate aminotransferase, ALT = alanine aminotransferase, GGT = gamma-glutamyl transferase.

a Adjusted for age and sex, smoking, moderate/vigorous intensity exercise, income level, insurance, residence, fasting blood glucose, high blood pressure, and dyslipidemia.

**Table 5.** Joint and additive interaction effects of binge drinking and abdominal obesity on elevated liver enzymes

Outcome	BD intensity	Abdominal obesity				Interaction measures		
		No		Yes		RERI^a^ (95% CI)	AP^a^ (95% CI)	SI^a^ (95% CI)
		*n*	OR^a^ (95% CI)	*n*	OR^a^ (95% CI)			
Elevated ALT								
	Non-BD	4,364	1.00	2,589	2.77 (2.62–2.93)	0.16 (–0.03–0.36)	0.05 (–0.02–0.13)	1.10 (0.98–1.21)
	BD I	2,384	1.11 (1.06–1.18)	1,643	3.06 (2.86–3.27)			
	HIBD	683	1.14 (1.05–1.25)	582	3.04 (2.74–3.36)			
Elevated AST								
	Non-BD	10,777	1.00	4,834	2.70 (2.59–2.83)	0.25 (0.10–0.42)	0.10 (0.01–0.14)	1.13 (1.04–1.24)
	BD I	5,776	1.14 (1.10–1.18)	3,017	3.10 (2.93–3.28)			
	HIBD	1,695	1.23 (1.16–1.31)	1,074	3.16 (2.88–3.45)			
Elevated GGT								
	Non-BD	10,790	1.00	3,953	1.75 (1.67–1.83)	0.79 (0.62–0.95)	0.24 (0.19–0.29)	1.56 (1.42–1.70)
	BD I	6,898	1.62 (1.56–1.68)	3,013	3.09 (2.92–3.27)			
	HIBD	2,008	1.88 (1.77–1.99)	1,101	3.62 (3.31–3.97)			

BD = binge drinking, HIBD = high-intensity binge drinking, BMI = body mass index, RERI = relative excess risk due to interaction, AP = attributable proportion, SI = synergy index, BD I = level I binge drinking, AST = aspartate aminotransferase, ALT = alanine aminotransferase, GGT = gamma-glutamyl transferase.

a Adjusted for age and sex, smoking, moderate/vigorous intensity exercise, income level, insurance, residence, fasting blood glucose, high blood pressure, and dyslipidemia.

### Additive interaction measures for the joint effects between obesity and BD on elevated liver enzymes

The excess risks of elevated AST and GGT explained by the interaction between BD and BMI were 23% and 67%, respectively. In addition, the proportions of elevated AST and GGT attributable to the interaction between BD and BMI were 7% and 24%, respectively. Consistently, the synergistic effects of BD and BMI on elevated AST and GGT were 13% and 58%, respectively (Table 4). Similar findings were observed with BD and abdominal obesity. The RERI, AP, and SI for the interaction effects between BD and abdominal obesity were 25%, 10%, and 13% for elevated AST, and 79%, 24%, and 56% for elevated GGT, respectively (Table 5).

## Discussion

The prevalence of BD in Korea is high, previously estimated at 46% and 9.2% in men and women, respectively [2]. However, there are few reports on the health implications of this behavior in the Korean population. This cross-sectional analysis of the 2009–2015 KNHIS data investigated the independent and joint association of BD and obesity with elevated liver enzymes. Results showed that HIBD and general and abdominal obesity were independently and positively associated with high odds of elevated liver enzymes. Moreover, positive synergistic effects of BD and general or abdominal obesity on elevated AST and GGT were observed.

Few population-based studies have investigated the implications of BD on liver enzymes and none has been conducted in Korea. Consistent with our findings, HIBD was associated with elevated liver enzymes [16]. BD once a month was associated with a significant increase in GGT and ALT levels, even among low habitual alcohol consumers [17], and an experimental study showed that BD increased liver enzymes that promote oxidative and pro-inflammatory states, and potential progression to advanced liver disease [33]. Moreover, BD was positively associated with obesity and obesity-related comorbidities in the Korean population [34]. The independent effects of obesity on liver enzymes have been reported previously. Obesity is independently associated with elevated liver enzymes [13, 21, 35–37], with a 2- to 3-fold increase in the risk of elevated liver enzymes [38].

Our study suggests that BD and obesity are independently associated with increased liver enzyme activity. The clinical relevance of liver enzymes is broadly supported by previous studies from diverse populations, which reported that elevated liver enzymes above normal ranges defined in our study were positively associated with all-cause, liver disease, and liver cancer mortality [39–42]. Therefore, increased liver enzymes above normal ranges may act as early biomarkers of liver injury [43]. Since liver disease is often diagnosed at advanced stages, understanding modifiable lifestyle predictors of elevated liver injury biomarkers is relevant for identifying individuals who may be at risk of future disease and targeting them for lifestyle interventions. Lifestyle interventions, such as diet-induced weight loss, have been shown to improve liver enzyme levels in individuals with obesity [44, 45].

Previous studies that investigated the joint effects of alcohol intake and adiposity on hepatic health have mostly focused on chronic alcohol intake. Increased adiposity together with moderate or heavy alcohol intake synergistically promote elevated liver enzymes [18, 19, 21–23] and increase the risk of liver disease [21, 43, 46] and liver disease-related mortality [43, 46]. To our knowledge, our study is the first to evaluate biologically meaningful interactions between adiposity and BD on markers of hepatic health.

Our findings are supported by mechanistic studies. Chang *et al.* [41] showed that BD and high-fat feeding synergistically induced steatohepatitis and fibrosis via elevation of liver or serum free fatty acids, upregulation of chemokine ligand 1 (CXCL1) expression, and promotion of hepatic neutrophil infiltration [47, 48]. Both ethanol and adiposity, especially abdominal fat, may cause fatty liver via a pro-inflammatory state that induces tumor necrosis factor (TNF) activity and hepatic insulin resistance. A combination of reduced serum adiponectin concentration and high TNF activity associated with both ethanol and visceral adiposity promotes steatohepatitis [49].

The major strengths of this study include use of a large sample size from a nationally representative sample of the Korean population, formal statistical evaluation of biologically meaningful interactions [32], evaluation of both general and abdominal obesity independently and jointly with BD in relation to liver function markers, and adjustment of potential confounders in the models. However, potential limitations should be acknowledged. First, the cross-sectional design precludes evaluation of causal relationships. However, Mendelian randomization analyses showed that obesity and high alcohol intake may be causally related to elevated circulating ALT and GGT [21, 50]. Second, self-desirability and recall bias in self-reported alcohol intake could have led to underestimation of the proportion of BD and potential underestimation of ORs. Finally, the KNHIS is an administrative database based on the Korean population. The overall results may have been contaminated by coding inaccuracies and may not be representative of other Asian populations.

## Conclusions

BD and obesity independently and synergistically elevated circulating liver enzymes, suggesting that participants with obesity who engage in BD may be more susceptible to liver injury. Our results provided basic data to inform the design of prospective cohort studies to investigate whether high episodic drinking and obesity synergistically increased the risk of clinical liver disease and related mortality. Meanwhile, the data supported the need for public health campaigns that emphasize the reduction of alcohol intake and adoption of behaviors for weight reduction.

## Supplementary Material

goad074_Supplementary_Data
