# Supplementary material for: Independent and additive effects of binge drinking and obesity on liver enzymes: a cross-sectional analysis using the Korean National Health Insurance Service data
Source: Gastroenterol Rep (Oxf). 2024 Jan 9;12:goad074. doi: 10.1093/gastro/goad074 (PMC10784631; doi:10.1093/gastro/goad074)
Supplement: goad074_Supplementary_Data [file goad074_supplementary_data.zip › List of References.docx]

**References**

1. WHO. The Global Health Observatory. Alcohol, total per capita (15+) consumption (in litres of pure alcohol) (SDG Indicator 3.5.2) [Internet]. 2022 [Accessed 2022 Sep 9]. Available from: https://www.who.int/data/gho/data/indicators/indicator-details/GHO/total-(recorded-unrecorded)-alcohol-per-capita-(15-)-consumption

2. Sull JW, Yi S-W, Nam CM, et al. Binge Drinking and Hypertension on Cardiovascular Disease Mortality in Korean Men and Women: A Kangwha Cohort Study. *Stroke*. 2009;**40**(9):2953–8.

3. Kim HN, Song SW. Relationships of both Heavy and Binge Alcohol Drinking with Unhealthy Habits in Korean Adults Based on the KNHANES IV Data. *Iran J Public Health*. 2014;**43**(5):579-589.

4. Graff-Iversen S, Jansen MD, Hoff DA, et al. Divergent associations of drinking frequency and binge consumption of alcohol with mortality within the same cohort. J Epidemiol Community Health. 2013;**67**(4):350–357.

5. Horvat P, Stefler D, Murphy M, et al. Alcohol, pattern of drinking and all-cause mortality in Russia, Belarus and Hungary: a retrospective indirect cohort study based on mortality of relatives. *Addiction*. 2018;**113**(7):1252–1263.

6. Sundell L, Salomaa V, Vartiainen E, et al. Increased stroke risk is related to a binge-drinking habit. *Stroke*. 2008;**39**(12):3179–3184.

7. Kuntsche E, Kuntsche S, Thrul J, et al. Binge drinking: Health impact, prevalence, correlates and interventions. *Psychol Health*. 2017;**32**(8):976–1017.

8. WHO. Global status report on alcohol and health 2018. [Internet]. 2018 [Accessed 2022 Sep 9]. Available from: <https://www.who.int/publications/i/item/9789241565639>

9. Han M, Lee S-R, Choi E-K, et al. Habitual Alcohol Intake and Risk of Atrial Fibrillation in Young Adults in Korea. *JAMA Netw Open*. 2022;**5**(9):e2229799–e2229799.

10. Kim YG, Han K, Choi JI, et al. Frequent drinking is a more important risk factor for new-onset atrial fibrillation than binge drinking: A nationwide population-based study. *Europace.* 2020;**22**(2):216–224.

11. Åberg F, Helenius-Hietala J, Puukka P, et al. Binge drinking and the risk of liver events: A population-based cohort study. *Liver Int.* 2017;**37**(9):1373–1381.

12. Li L, Liu DW, Yan HY, et al. Obesity is an independent risk factor for non-alcoholic fatty liver disease: evidence from a meta-analysis of 21 cohort studies. *Obes Rev*. 2016;**17**(6):510–519.

13. Parker R, Kim SJ, Gao B. Alcohol, adipose tissue and liver disease: mechanistic links and clinical considerations. *Nat Rev Gastroenterol Hepatol*. 2018;**15**(1):50–59.

14. Park SH, Plank LD, Suk KT, et al. Trends in the prevalence of chronic liver disease in the Korean adult population, 1998-2017. *Clin Mol Hepatol*. 2020;**26**(2):209–215.

15. Harris JC, Leggio L, Farokhnia M. Blood Biomarkers of Alcohol Use: A Scoping Review. *Curr Addict Rep*. 2021;**8**(4):500–508.

16. Park EO, Bae EJ, Park BH, et al. The Associations between Liver Enzymes and Cardiovascular Risk Factors in Adults with Mild Dyslipidemia. *J Clin Med*. 2020;**9**(4):1147

17. Adams LA, Knuiman MW, Divitini ML, et al. Body mass index is a stronger predictor of alanine aminotransaminase levels than alcohol consumption. *J Gastroenterol Hepatol*. 2008;**23**(7 Pt 1):1089–1093.

18. Kunutsor SK, Apekey TA, Khan H. Liver enzymes and risk of cardiovascular disease in the general population: A meta-analysis of prospective cohort studies. *Atherosclerosis*. 2014;**236**(1): 7-17.

19. Unalp-Arida A, Ruhl CE. Noninvasive fatty liver markers predict liver disease mortality in the U.S. population. *Hepatology*. 2016;**63**(4):1170–1183.

20. Rosoff DB, Charlet K, Jung J, et al. Association of High-Intensity Binge Drinking With Lipid and Liver Function Enzyme Levels. *JAMA Netw Open*. 2019;**2**(6):e195844–e195844.

21. Nivukoski U, Bloigu A, Bloigu R, et al. Liver enzymes in alcohol consumers with or without binge drinking. *Alcohol*. 2019;**78**:13–19.

22. Loomba R, Bettencourt R, Barrett-Connor E. Synergistic association between alcohol intake and body mass index with serum alanine and aspartate aminotransferase levels in older adults: the Rancho Bernardo Study. *Aliment Pharmacol Ther*. 2009;**30**(11–12):1137–1149.

23. Shen Z, Li Y, Yu C, et al. A cohort study of the effect of alcohol consumption and obesity on serum liver enzyme levels. *Eur J Gastroenterol Hepatol*. 2010;**22**(7):820–825.

24. Ruhl CE, Everhart JE. Joint Effects of Body Weight and Alcohol on Elevated Serum Alanine Aminotransferase in the United States Population. *Clin Gastroenterol Hepatol.* 2005;**3**(12):1260–1268.

25. Carter AR, Borges MC, Benn M, et al. Combined Association of Body Mass Index and Alcohol Consumption With Biomarkers for Liver Injury and Incidence of Liver Disease: A Mendelian Randomization Study. *JAMA Netw Open*. 2019;**2**(3): e190305.

26. Puukka K, Hietala J, Koivisto H, Anttila P, Bloigu R, Niemelä O. Additive effects of moderate drinking and obesity on serum γ-glutamyl transferase activity. *Am J Clin Nutr*. 2006;**83**(6):1351–1354.

27. Alatalo PI, Koivisto HM, Hietala JP, et al. Effect of moderate alcohol consumption on liver enzymes increases with increasing body mass index. *Am J Clin Nutr*. 2008;**88**(4):1097–103.

28. National Health Insurance Service [Internet]. [Accessed 2022 Sep 7]. Available from: <https://nhiss.nhis.or.kr/>

29. WHO. International Guide for Monitoring Alcohol Consumption and Related Harm [Internet]. Geneva; 2000 [Accessed 2022 Sep 7]. Available from: <https://www.who.int/publications/i/item/international-guide-for-monitoring-alcohol-consumption-and-related-harm>

30. Hingson RW, Zha W, White AM. Drinking Beyond the Binge Threshold: Predictors, Consequences, and Changes in the U.S. *Am J Prev Med*. 2017;**52**(6):717–727.

31. Patrick ME. A Call for Research on High-Intensity Alcohol Use. *Alcohol Clin Exp Res*. 2016;**40**(2):256–259.

32. Pan W-H, Yeh W-T. How to define obesity? Evidence-based multiple action points for public awareness, screening, and treatment: an extension of Asian-Pacific recommendations. *Asia Pac J Clin Nutr*. 2008;**17**(3):370-374.

33. Alberti KGMM, Zimmet P, Shaw J. Metabolic syndrome - A new world-wide definition. A consensus statement from the International Diabetes Federation. *Diabet Med*. 2006;**23**(5):469–480.

34. Kwo PY, Cohen SM, Lim JK. ACG Clinical Guideline: Evaluation of Abnormal Liver Chemistries. *Am J Gastroenterol*. 2017;**112**(1):18–35.

35. Hosmer DW, Lemeshow S. Confidence interval estimation of interaction. *Epidemiology*. 1992;**3**(5):452–456.

36. Andersson T, Alfredsson L, Källberg H, et al. Calculating measures of biological interaction. *Eur J Epidemiol*. 2005;**20**(7):575–579.

37. Wegner SA, Pollard KA, Kharazia V, et al. Limited Excessive Voluntary Alcohol Drinking Leads to Liver Dysfunction in Mice. *Alcohol Clin Exp Res*. 2017;**41**(2):345–358.

38. Kim BY, Nam H, Yoo JJ, et al. Association between alcohol consumption status and obesity-related comorbidities in men: data from the 2016 Korean community health survey. *BMC Public Health*. 2021;**21**(1):733.

39. Liu C, Shao M, Lu L, et al. Obesity, insulin resistance and their interaction on liver enzymes. *PLoS One*. 2021;**16**(4): e0249299.

40. Marchesini G, Avagnina S, Barantani EG, et al. Aminotransferase and gamma-glutamyltranspeptidase levels in obesity are associated with insulin resistance and the metabolic syndrome. *J Endocrinol Invest*. 2005;**28**(4):333–339.

41. Ali N, Sumon AH, Fariha KA, et al. Assessment of the relationship of serum liver enzymes activity with general and abdominal obesity in an urban Bangladeshi population. *Sci Rep*.2021;**11**(1):1–9.

42. Marchesini G, Avagnina S, Barantani EG, et al. Aminotransferase and gamma-glutamyltranspeptidase levels in obesity are associated with insulin resistance and the metabolic syndrome. *J Endocrinol Invest*. 2005;**28**(4):333–339.

43. Sinn DH, Kang D, Guallar E, et al. Modest alcohol intake and mortality in individuals with elevated alanine aminotransferase levels: a nationwide cohort study. *BMC Med*. 2022;**20**(1):18.

44. Kim KN, Joo J, Sung HK, et al. Associations of serum liver enzyme levels and their changes over time with all-cause and cause-specific mortality in the general population: a large-scale national health screening cohort study. *BMJ Open*. 2019;**9**(5): e026965.

45. Xie K, Chen CH, Tsai SP, et al. Loss of Life Expectancy by 10 Years or More From Elevated Aspartate Aminotransferase: Finding Aspartate Aminotransferase a Better Mortality Predictor for All-Cause and Liver-Related than Alanine Aminotransferase. *Am J Gastroenterol*. 2019;**114**(9):1478–1487.

46. Katzke V, Johnson T, Sookthai D, Hüsing A, Kühn T, Kaaks R. Circulating liver enzymes and risks of chronic diseases and mortality in the prospective EPIC-Heidelberg case-cohort study. BMJ Open. 2020;10(3).

47. Katzke V, Johnson T, Sookthai D, et al. Circulating liver enzymes and risks of chronic diseases and mortality in the prospective EPIC-Heidelberg case-cohort study. *BMJ Open*. 2020;**10**(3): e033532.

48. Gasteyger C, Larsen TM, Vercruysse F, et al. Effect of a dietary-induced weight loss on liver enzymes in obese subjects. *Am J Clin Nutr*. 2008;**87**(5):1141–1147.

49. Straznicky NE, Lambert EA, Grima MT, et al. The effects of dietary weight loss with or without exercise training on liver enzymes in obese metabolic syndrome subjects. *Diabetes Obes Metab*. 2012;**14**(2):139–148.

50. Inan-Eroglu E, Huang BH, Ahmadi MN, et al. Joint associations of adiposity and alcohol consumption with liver disease-related morbidity and mortality risk: findings from the UK Biobank. *Eur J Clin Nutr*. 2022;**76**(1):74–83.

51. Chang B, Xu MJ, Zhou Z, et al. Short- or long-term high fat diet feeding plus acute ethanol binge synergistically induce acute liver injury in mice: an important role for CXCL1. *Hepatology*. 2015;**62**(4):1070-1085.

52. Hwang S, Ren T, Gao B. Obesity and binge alcohol intake are deadly combination to induce steatohepatitis: A model of high-fat diet and binge ethanol intake. *Clin Mol Hepatol*. 2020;**26**(4):586–594.

53. Diehl AM. Obesity and alcoholic liver disease. *Alcohol*. 2004;**34**(1):81–87.

54. Lawlor DA, Benn M, Zuccolo L, et al. ADH1B and ADH1C genotype, alcohol consumption and biomarkers of liver function: Findings from a Mendelian randomization study in 58,313 European origin Danes. *PLoS One*. 2014;**9**(12): e114294.
