# Supplementary material for: Independent and additive effects of binge drinking and obesity on liver enzymes: a cross-sectional analysis using the Korean National Health Insurance Service data
Source: Gastroenterol Rep (Oxf). 2024 Jan 9;12:goad074. doi: 10.1093/gastro/goad074 (PMC10784631; doi:10.1093/gastro/goad074)
Supplement: goad074_Supplementary_Data [file goad074_supplementary_data.zip › Supplementary_Tables.docx]

**Independent and additive effects of binge drinking and obesity on liver enzymes:** **a cross-sectional analysis using the Korean national health insurance service data**

**Anthony Kityo and Sang-Ah Lee**

**Supplementary Tables**

Table 1. Prevalence of elevated liver enzymes according to binge drinking intensity

| Characteristics | Binge drinking intensity | | | |
| --- | --- | --- | --- | --- |
|  | Never drinker  (*n* = 136,715) | Non-BD  (*n* = 93,536) | BD I  (*n* = 43,171) | HIBD  (*n* = 12,178) |
| Moderate physical exercise, % |  |  |  |  |
| Yes | 59021 (43.2) | 47886 (51.2) | 21669 (50.2) | 6093 (50.0) |
| No | 77694 (56.8) | 45650 (48.8) | 21502 (49.8) | 6085 (50.0) |
| Abdominal obesity, % |  |  |  |  |
| Yes | 29413 (21.5) | 14762 (15.8) | 7889 (18.3) | 2636 (21.7) |
| No | 107302 (78.5) | 78774 (84.2) | 35282 (81.7) | 9542 (78.4) |
| Elevated ALT, % |  |  |  |  |
| Yes | 10,196 (7.5) | 6,953 (7.4) | 4,027 (9.3) | 1,265(10.4) |
| No | 126519 (92.5) | 86583 (92.6) | 39144 (90.7) | 10913 (89.6) |
| Elevated AST, % |  |  |  |  |
| Yes | 16,470 (12.1) | 15611 (16.7) | 8793 (20.4) | 2769 (22.7) |
| No | 120245 (87.95) | 77925 (83.31) | 34378 (79.6) | 9409 (77.3) |
| Elevated GGT, % |  |  |  |  |
| Yes | 7970 (5.8) | 14743 (15.8) | 9911 (23.0) | 3109 (25.5) |
| No | 128745 (94.2) | 78793 (84.2) | 33260 (77.0) | 9069 (74.5) |
| Dyslipidemia, % |  |  |  |  |
| Yes | 58722 (43.0) | 37050 (39.6) | 18426 (42.7) | 5311 (43.6) |
| No | 77993 (57.1) | 56486 (60.4) | 24745 (57.3) | 6867 (56.4) |
| High blood pressure, % |  |  |  |  |
| Yes | 35597 (26.0) | 30422 (32.5) | 15270 (35.4) | 4344 (35.7) |
| No | 101118 (74.0) | 63114 (67.5) | 27901 (64.6) | 7834 (64.3) |

BD, binge drinking; HIBD, high intensity binge drinking; AST, Aspartate aminotransferase; ALT, Alanine aminotransferase; GGT, gamma-glutamyl transferase.

Table 2. Prevalence of elevated liver enzymes according to general and abdominal obesity

|  | BMI category, kg/m^2^ | | | |  | Abdominal obesity | |
| --- | --- | --- | --- | --- | --- | --- | --- |
| Characteristic | <18.0  (*n* = 13,657) | ≤22.9  (*n* = 121,780) | ≤24.9  (*n* = 65,943) | ≥25.0  (*n* = 84,220) |  | No  (*n* = 230,900) | Yes  (*n* = 54,700) |
| Moderate physical exercise, % |  |  |  |  |  |  |  |
| Yes | 5,698 (41.7) | 56,535 (46.4) | 32,021 (48.6) | 40,415 (48.0) |  | 110,041 (47.7) | 24,628 (45.0) |
| No | 7,959 (58.3) | 65,245 (53.6) | 33,922 (51.4) | 43,805 (52.0) |  | 120,859 (52.3) | 30,072 (55.0) |
| Elevated ALT, % |  |  |  |  |  |  |  |
| Yes | 303 (2.2) | 4,145 (3.4) | 4,499 (6.8) | 13,494 (16.0) |  | 13,102 (5.7) | 9,339 (17.1) |
| No | 13,354 (97.8) | 117,635 (96.6) | 61,444 (93.2) | 70,726 (84.0) |  | 217,798 (94.3) | 45,361 (82.9) |
| Elevated AST, % |  |  |  |  |  |  |  |
| Yes | 459 (3.4) | 8,120 (6.7) | 9,824 (14.9) | 25,240 (30.0) |  | 28,419 (12.3) | 15,224 (27.8) |
| No | 13,198 (96.6) | 113,660 (93.3) | 56,119 (85.1) | 58,980 (70.0) |  | 202,481 (87.7) | 39,476 (72.2) |
| Elevated GGT, % |  |  |  |  |  |  |  |
| Yes | 493 (3.6) | 8,045 (6.6) | 8,583 (13.0) | 18,612 (22.1) |  | 24,597 (10.7) | 11,136 (20.4) |
| No | 13,164 (96.4) | 113,735 (93.4) | 57,360 (87.0) | 65,608 (77.9) |  | 206,303 (89.4) | 43,564 (79.6) |
| Dyslipidemia, % |  |  |  |  |  |  |  |
| Yes | 2,167 (15.9) | 35,825 (29.4) | 30,726 (46.6) | 50,791 (60.3) |  | 85,622 (37.1) | 33,887 (62.0) |
| No | 11,490 (84.1) | 85955 (70.6) | 35217 (53.4) | 33429 (39.7) |  | 145278 (62.9) | 20813 (38.1) |
| High blood pressure, % |  |  |  |  |  |  |  |
| Yes | 1,467 (10.7) | 24450 (20.1) | 21357 (32.4) | 38359 (45.6) |  | 61070 (26.5) | 24563 (44.9) |
| No | 12,190 (89.3) | 97330 (79.9) | 44586 (67.6) | 45861 (54.5) |  | 169830 (73.6) | 30137 (55.1) |

AST, Aspartate aminotransferase; ALT, Alanine aminotransferase; GGT, gamma-glutamyl transferase.

Table 3. Independent association of Binge drinking, and obesity measures with elevated liver function enzymes

|  | Elevated liver enzymes | | | | | | | |
| --- | --- | --- | --- | --- | --- | --- | --- | --- |
|  | ALT | |  | AST | |  | GGT | |
|  | OR^a^ (95% CI) | OR^b^ (95% CI) |  | OR (95% CI) | OR (95% CI) |  | OR (95% CI) | OR (95% CI) |
| Binge drinking intensity |  |  |  |  |  |  |  |  |
| Non-BD | 0.81 (0.78-0.83) | 0.79 (0.76-0.82) |  | 0.87 (0.85-0.89) | 0.89 (0.87-0.92) |  | 2.15 (2.09-2.22) | 2.21 (2.14-2.28) |
| Never drinker | 1.00 | 1.00 |  | 1.00 | 1.00 |  | 1.00 | 1.00 |
| BD II | 1.06 (1.01-1.1) | 1.01 (0.97-1.05) |  | 1.14 (1.11-1.18) | 1.04 (1.01-1.07) |  | 3.99 (3.85-4.13) | 3.67 (3.54-3.81) |
| HIBD | 1.21 (1.14-1.29) | 1.14 (1.07-1.21) |  | 1.34 (1.28-1.41) | 1.08 (1.03-1.14) |  | 5.04 (4.79-5.3) | 4.21 (3.99-4.43) |
|  |  |  |  |  |  |  |  |  |
| Body mass index |  |  |  |  |  |  |  |  |
| <18.0 | 0.68 (0.6-0.76) | 0.67 (0.6-0.76) |  | 0.62 (0.56-0.68) | 0.61 (0.55-0.67) |  | 0.75 (0.68-0.82) | 0.75 (0.68-0.82) |
| 18.0-22.9 | 1.00 | 1.00 |  | 1.00 | 1.00 |  | 1.00 | 1.00 |
| 23.0-24.9 | 1.97 (1.88-2.06) | 1.98 (1.89-2.07) |  | 2 (1.94-2.07) | 2.02 (1.96-2.09) |  | 1.6 (1.55-1.66) | 1.65 (1.6-1.71) |
| ≥25.0 | 5.05 (4.87-5.24) | 5.04 (4.86-5.23) |  | 4.82 (4.69-4.95) | 4.82 (4.69-4.96) |  | 2.99 (2.9-3.07) | 3.09 (3-3.18) |
|  |  |  |  |  |  |  |  |  |
| WC |  |  |  |  |  |  |  |  |
| No | 1.00 | 1.00 |  | 1.00 | 1.00 |  | 1.00 | 1.00 |
| Yes | 3.71 (3.61-3.82) | 3.66 (3.56-3.77) |  | 3.58 (3.49-3.67) | 3.53 (3.44-3.61) |  | 2.57 (2.5-2.64) | 2.57 (2.5-2.64) |

BD, binge drinking; HIBD, high intensity binge drinking; AST, Aspartate aminotransferase; ALT, Alanine aminotransferase; GGT, gamma-glutamyl transferase,

^a^ Adjusted for age and sex

^b^ Adjusted for age, sex, smoking, drinking, moderate/vigorous intensity exercise, income level, insurance, and residence
